# Molecular identification and successful treatment of *Chlamydophila psittaci* (genotype B) in a clinically affected Congo African grey parrot (*Psittacus erithacus erithacus*)

**Published:** 2016

**Authors:** J. Razmyar, M. Rajabioun, M. Zaeemi, A. Afshari

**Affiliations:** 1 Department of Clinical Sciences, Faculty of Veterinary Medicine, Ferdowsi University of Mashhad, Mashhad, Iran;; 2 Department of Nutrition, Faculty of Medicine, Mashhad University of Medical Science, Mashhad, Iran

**Keywords:** Avian chlamydiosis, *Chlamydophila psittaci* genotype B, Congo African grey parrot, *ompA* gene, PCR

## Abstract

Avian chlamydiosis is caused by *Chlamydiophila psittaci* with the highest infection rate in parrots (*Psittacidae*) and pigeons (*Columbiformes*). A two-year-old Congo African grey parrot was examined since the bird had shown clinical signs of anorexia, depression, diarrhea, and mild dyspnea and based on biochemical and hemathological analysis the bird was diagnosed as having anemia, leukocytosis, heterophilia, lymphopenia and monocytosis. With regards to clinical and paraclinical findings, the case was diagnosed to be carrying *Chlamydiophila* spp. In addition, choanal cleft and cloaca swabs were positive for *Chlamydiophila* spp. in a diagnostic polymerase chain reaction (PCR) (600 bp amplicon). Polymerase chain reaction products were typed by *ompA* gene-based PCR, using CTU/CTL primers (1050 bp amplicon). The PCR product sequence was compared with the sequences obtained from GenBank. The phylogenetic tree has revealed 100% identity with genotype B obtained from previous studies. The bird was hospitalized and treated with doxycycline regimen for 45 days, with a weekly sampling process to trace the presence of *C. psittaci* DNA in faecal and choanal swabs, this process continued to the point where the specimens turned negative after two weeks. Laboratory and radiology results were within normal limits after the treatment. Genotype B is predominantly isolated from *Columbidae* and there have not been any reports regarding the clinically affected African gray parrot with this genotype. Subsequently, to the best of our knowledge, this is the first report of chlamydiosis by genotype B on Congo African grey parrot.

## Introduction

Chlamydiosis is noteworthy in avian medicine because it is a zoonotic disease and also infected birds do not show specific clinical signs. Lethargy, anorexia, diarrhea, ocular or nasal discharge, and green to yellow-green urates are common clinical signs of avian chlamydiosis and severity of symptoms depends on different factors such as; the serotype of the bacterium, species, health and nutrition status of infected bird. Human infection occurred by inhalation of aerosolized bacteria or by exposure to contaminated faeces, feather or tissue. During immunosuppression status, subclinical carriers also shed bacteria through nasal discharge and faeces (West, ‎2011 ▶).

*Chlamydiophila psittaci*, the agent of avian chlamy-diosis, is an obligate intracellular gram-negative bacterium in the order *Chlamydiales*, family *Chlamydiaceae* that are widely distributed throughout the world. The family composed of a single genus, Chlamydia comprises 11 species: *C. abortus*, *C. avium*, *C. caviae*, *C. felis*, *C. gallinacea*, *C. muridarum*, *C. pecorum*, *C. pneumoniae*, *C. psittaci*, *C. suis*, and *C. trachomatis* (Schachter, 1999 ▶).

This bacterium colonizes a wide range of hosts *C. psittaci* is the primary avian pathogen that can infect a large number of bird species (it has been isolated from more than 450 avian species), as well as, mammals (Kaleta and Taday, 2003 ▶).

Avian strains of *C. psittaci* are currently divided into at least 15 genotypes based on outer membrane protein A gene (*ompA*) (Madani and Peighambari, 2013 ▶), reports shown that each genotype tending to be species-specific (Kaleta and Taday, 2003 ▶).

In the present study, we found a rare condition, an African grey parrot affected with genotype B, that mainly frequent in pigeon populations.

## Case presentation

A two-year-old Congo African grey parrot was presented to the Veterinary Teaching Hospital, Ferdowsi University of Mashhad with onset of anorexia, depression, diarrhea, and mild dyspnea. The bird had been housed in a private aviary where there had not been any direct contact with wild birds. For hematological analysis, blood sample was collected from brachial vein and for bacterial identification specimens were collected by taking triple samples of the bird’s choanal cleft and cloaca, using single sterile swabs. Nasal and pharyngeal swabs from two owners were also collected. Swabs were immersed in 1 ml SPG (Sucrose-Phosphate-Glutamic acid) buffer (Merck-Germany) and refrigerated at 4°C overnight and then centrifuged at 500 × g for 20 min. After double centrifugation, the final supernatant was used for DNA extraction (OIE, 2008 ▶).

The packed cell volume (PCV) was obtained by using micro hematocrit technique. The total white blood cell (WBC) count was determined by Natt and Herrick technique (NHT) (Natt and Herrick, 1952 ▶). The differential cell count was performed on blood smear stained by Giemsa solution (5%). Serum biochemical variables were determined by an auto-analyzer (Biotecnica, BT1500, Rome, Italy) along with the commercial kits (Pars Azmoon, Iran). The accuracy of measurements was checked by control serum (Randox control sera, Antrim, UK). The serum concentration of globulin was calculated by subtraction of albumin of the total protein. Lateral and ventrodorsal (VD) radiograph was obtained using mammography cassette to yield the optimal quality with exposure factors set at 48 kVp and 8 mAs. Radiography was repeated 45 days after the treatment in order to evaluate the liver and the kidneys (Siemens MULTIX TOP X-ray machine, Germany).

The treatment was provided by administrating 25 mg/kg (1 g/L of 20%) of doxycycline (Tolide Darouhai Dami Iran Co., Iran) every 24 h for 45 days. For DNA extraction, PCR Preparation kit (Denazist Asia, Iran) was used, according to the manufacturer’s instructions.

The extracted DNA was analyzed by a diagnostic polymerase chain reaction (PCR) according to Sachse and Hotzel (2003) ▶. The primers 191CHOMP 5´-GCI YTI TGG GAR TGY GGI TGY GCI AC-3´ and CHOMP371 5´-TTA GAA ICK GAA TTG IGC RTT IAY GTG IGC IGC-3´ targeted *ompA* gene. The reaction mixture and PCR are similar to PCR for sequencing analysis (1050 bp). For sequencing assay we have targeted the 1050 bp DNA fragment of *ompA* gene by using CTU (5´-ATG AAA AAA CTC TTG AAA TCG G-3´) and CTL (5´-CAA GAT TTT CTA GA (T/C) TTC AT (C/T) TTG-3´) primers (Denazist Asia, Iran). The reaction mixture and PCR reactions conditions for both PCRs were performed according to Sache et al. (2008) ▶ with using only one set of primers as a diagnostic PCR. Amplification reactions were carried out in thermocycler (Techne TC, 3000, England). The DNA was electrophoresed in 1% agarose gel containing ethidium-bromide (Sachse et al., 2008 ▶).

The PCR product (1050 bp) was purified and submitted for automated sequencing in both directions at the Macrogen (South Korea) using PCR primers as sequencing primers. Nucleotide and predicted amino acid sequence data were aligned with the clustal alignment algorithm. Phylogenetic analysis was based on both nucleotide and predicted amino acid sequence which was conducted by distance method, unweighted-pair group method with arithmetic means (UPGMA). The bootstrap values were calculated for 1000 replicates in CLC Work Bench Package Version 5 (CLC Bio, Aarhus, Denmark). The sequence data was submitted to GenBank under the accession number; KT223044. The accession numbers of reference genotype’s sequences used for multiple alignment analysis are shown in Table 1.

## Results

Hematological analysis indicated anemia, leuko-cytosis, heterophilia, lymphopenia and monocytosis. Leukocytosis and anaemia were improved during 45 days of doxycycline treatment. Liver enzyme activities and protein profile were in normal reference range after the treatment (Table 2).

**Table 2:** Biochemical parameters and reference ranges for Congo African gray parrot before and after treatment (Fudge, 2000

Parameters	Before treatment	After treatment	References values (Fudge, 2000)
PCV (%)	24	46	45-53
WBC (×10^3^/µL)	21.3	10	6-13
Heterophil (%)	82	65	45-73
Lymphocyte (%)	15	20	19-50
Monocyte (%)	3	2	0-2
Total protein (g/L)	70	40	27-44
Albumin (g/L)	20	22	20-24
Globulin (g/L)	50	35	12-36
A:G	0.4	0.62	0.42
Cholesterol (mg/dL)	402	350	100-250
AST (IU/L)	527	320	110-340
GGT (IU/L)	3	3	0-4
ALP (IU/L)	69	68	12-92
LDH (IU/L)	534	400	154-378
CK (IU)	389	390	140-411
Uric acid (mg/dL)	6.3	6	2-11

**Table 1:** Accession numbers of *C. psittaci* and *C. caviae OmpA* gene sequences used in this study

Species	Genotype	Strain	Accession No.	Reference
*C. psittaci*	B	41A12	AY762609	Geens *et al*. (2005)
*C. psittaci*	E/B	WS/RT/E30	AY762613	Geens *et al*. (2005)
*C. psittaci*	M56	–	AF269268	Bush and Everett (2001)
*C. psittaci*	F	VS225	AF269259	Bush and Everett (2001)
*C. psittaci*	C	–	L25436	Sachse (2008)
*C. psittaci*	D	NJ1	AF269266	Bush and Everett (2001)
*C. psittaci*	WC	–	AF269269	Bush and Everett (2001)
*C. psittaci*	E	EAE A22/M	X12647.1	Pickett *et al*. (1988)
*C. psittaci*	Provisional I	–	HQ845540	Madani and Peighambari (2013)
*C. psittaci*	Provisional J	UT78	HQ845545	Madani and Peighambari (2013)
*C. psittaci*	A	90/1051	AY762608	Geens *et al*. (2005)
*C. caviae*	–	GPIC	AF269282	Zhang *et al*. 1989; Bush and Everett (2001)

**Fig. 1:**
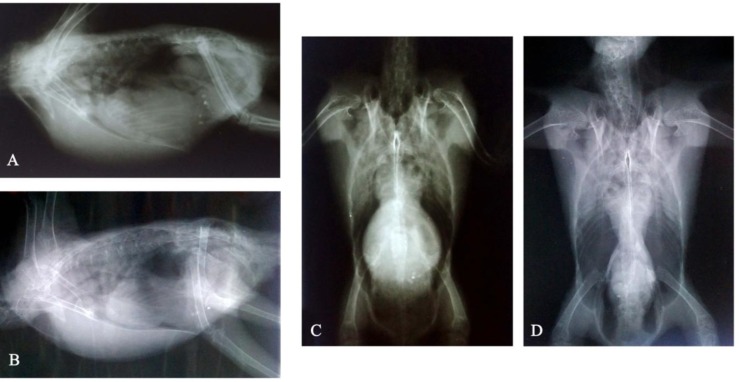
Lateral and ventrodorsal radiograph before (A and C, respectively) and after (B and D, respectively) treatment. Hepatomegaly and renal enlargement was seen in A and C. In B and C the kidney and liver size are within normal limit

**Fig. 2:**
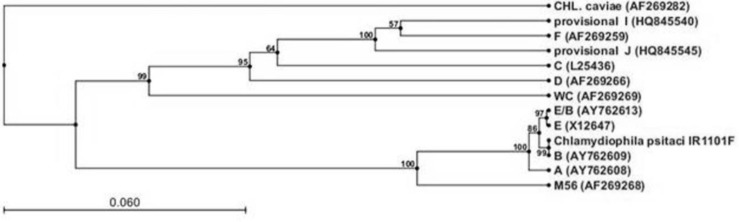
*OmpA* gene phylogenetic tree. Amino acid sequences of the *ompA* gene were used for phylogram construction. Note the neighbourhood of *C. psittaci* new genotype (IR1101F) and genotype B

Whole body lateral and VD radiograph showed hepatomegaly as well as increased kidney size (Figs. 1A and C). Triplicate samples of choanal cleft and cloaca showed the same PCR results and were positive at days 1, 7 and 14 after the 45-day doxycline treatment (Tolide Darouhai Dami Iran Co., Iran). The specimens turned negative in the second week (days 21 and 28) and the size of the liver was within normal limits and no sign of hepatomegaly and renal swelling was seen in the radiographic follow-up (Figs. 1B and D). The BLAST analysis of the *C. psittaci* IR110SR (GenBank: KT223044) shows 100% nucleotide and deduced amino acid sequences identity with *C. psittaci* 41A12 (GenBank AY762609), a genotype B isolate.

On the basis of 5% divergence criterium, 2 groups were identified in the established phylogenetic tree. The outgroup sequence belongs to a guinea pig strain of *C. caviae*. The first cluster was composed of three sub clusters (African grey parrot strain, Alexandrine Parakeet strain, orange-fronted parakeet strain, avian strain, turkey strain and cow strain of *C. psittaci*). Whereas the second cluster was composed of 2 sub clusters (duck strain, ewe strain, hares and muskrat strain and African grey parrot strain of *C. psittaci*) and current genotype and genotype B (strain 41A12) were placed in the third cluster (Fig. 2).

## Discussion

*Chlamydophila psittaci* is classified in *Chlamy-diaceae* and the genera *Chlamydophila* family. This bacterium could be classified into eight serovars (A to F, WC, and M56) by using monoclonal antibody specific for epitopes on the major outer membrane protein (*OMP*) and into nine genotypes (A to F, E/B, WC, and M56) based on *ompA* gene sequencing and gene analysis using CTU/CTL primers and *AluI* restriction enzyme (Geens et al., 2005 ▶). In this study, *C. psittaci* infection in a Congo African grey parrot was detected through evaluation of *ompA* gene in the samples by using PCR. The comparison of the nucleotide and deduced amino acid sequences of the *ompA* gene with other sequences available in the GenBank database, revealed a high degree of homology (100%) with *ompA* genes *C. psittaci* 41A12 (GenBank AY762609) genotype B. Madani and Peighambari (2013) ▶ identified genotype A in an African grey parrot and a lorikeet (*Trichoglossus haematodus*) while they had found genotype B in a rock dove (*Columbia livia*) and a canary (*Serinuscanaria*). Verification of *Chlamydophila psittasi* genotypes is especially important from the public health point of view, since all the genotypes could be transmitted to humans and could cause psittacosis or parrot fever (Andersen and Vanrompay, 2003 ▶). Genotype A is endemic among cockatoos, parrots, para-keets and Lories (*Psittaciformes*), and is a well-known zoonotic agent. However, genotype B is endemic among pigeons and has also been reported in psittacines and turkeys (Piasecki et al., 2012 ▶). Genotypes C and D are mostly detected in non-*Psittaciformes* (Andersen, 1997 ▶). They are highly pathogenic to domestic poultry, and are acknowledged occupational hazards for slaughterhouse workers and in general for everyone in contact with the poultry. In the current study, the genotype-B identification of a Congo African grey parrot indicated that cross-species transmission might have occurred previously because of the susceptibility of parrots to this genotype. Piasecki et al. (2012) ▶ investigated the prevalence of C. psittaci from 156 tracheal swab samples in 34 species of parrots in Poland. They reported genotype B in only two samples which had been raised in pigeon-shared aviaries.

Classic clinical pathologic abnormalities in chlamy-diosis include a non-regenerative anemia and severe leukocytosis with heterophilia and monocytosis on complete blood count (Campbell and Ellis, 2007 ▶). On a chemistry panel, the increase in aspartate amino-transferase, lactate dehydrogenase and bile acids are characteristic with or without an increase in uric acid (Fudge, 2000 ▶). Although renal swelling was observed in radiography, serum uric acid concentration was in reference range for African grey parrot which might be due to the concurrent decreased uric acid production caused by anorexia or liver disease (Fudge, 2000 ▶). Albumin concentration and A:G ratio were in lower limit of reference interval which was previously reported in *Psittacids* infected with *C. psittaci* (Harcourt-Brown and Chitty, 2005 ▶).

In this study *C. psittaci* was mainly found in cloacal swab, suggesting that Congo African grey parrot shed this bacterium in the environment via their faeces. The controversial results of psittacosis tests were negative in two persons involved with Congo African grey parrot, this might indicate low probability of direct contact of the owner with the bird, or intermittent shedding pattern of *C. psittaci* in faeces. There have been reports regarding genotype A zoonotic transmission from parrots, whereas genotype B strains are less virulent and less intensively excreted (Vanrompay et al., 1997). Recently, a large genomic analysis involving 20 *C. psittaci* genomes has shown that *C. psittaci* might have a history of frequently switching hosts because of its high rate of genetic recombination (Read et al., 2013 ▶), consequently, this explains genotype B detection in different hosts. In the Netherlands, genotype B was reported in three human cases with symptomatic psittacosis infection and is considered an underestimated source of the disease (Heddema et al., 2006 ▶). In another study, genotype B was discovered in nine human cases in Venezuela with symptomatic and asymptomatic infection, in which they were reported to have permanent pigeon presence in their vicinity (Arraiz et al., 2012 ▶).

Chlamydia seems to be difficult to eliminate from the environment, therefore this study suggests the need for greater awareness of chlamydiosis in pet bird populations by avian clinicians in Iran. Follow-up and treatment of confirmed cases along with detecting the agent in the persons in contact with the pet birds should be next on the agenda.

## References

[B1] Andersen AA (1997). Two new serovars of Chlamydia psittaci from North American birds. J. Vet. Diagnost. Inves.

[B2] Andersen AA, Vanrompay D (2003). Avian chlamydiosis (Psittacosis, Ornithosis). Diseases of poultry.

[B3] Arraiz, N, Bermudez, V, Urdaneta, B, Mujica, E, Sanchez, MP, Mejía, R, Prieto, C, Escalona, C, Mujica, A (2012). Evidence of zoonotic Chlamydophila psittaci transmission in a population at risk in Zulia state, Venezuela. Rev. Panam. Salud. Publica. (Bogota).

[B4] Bush RM, Everett K (2001). Molecular evolution of the chlamydiaceae. Int. J. Syst. Evol. Microbiol.

[B5] Campbell TW, Ellis CK (2007). Avian and exotic animal hematology and cytology..

[B6] Fudge AM, Fudge (2000). Avian liver and gastrointestinal testing. Laboratory medicine avian and exotic pets.

[B7] Geens T, Desplanques A, Van Loock M, Bönner BM, Kaleta EF, Magnino S, Andersen A, Everett KDA, Vanrompay D (2005). Sequencing of the Chlamydo-phila psittaci ompA gene reveals a new genotype, E/B, and the need for a rapid discriminatory genotyping method. J. Clin. Microbiol.

[B8] Harcourt-Brown N, Chitty J (2005). BSAVA manual of psittacine birds..

[B9] Heddema ER, Van Hannen EJ, Duim B, Vandenbroucke-Grauls CMJE, Pannekoek Y (2006). Genotyping of Chlamydophila psittaci in human samples. Emerg. Infect. Dis.

[B10] Kaleta EF, Taday EM (2003). Avian hosts range of Chlamydophila spp based on isolation, antigen detection, and serology. Avian Pathol.

[B11] Madani. SA, Peighambari SM (2013). PCR-based diagnosis, molecular characterization and detection of atypical strains of avian Chlamydia psittaci in companion and wild birds. Avian Pathol.

[B12] Natt MP, Herrick CA (1952). A new blood diluent for counting the erythrocytes and leucocytes of the chicken. Poult. Sci.

[B13] OIE A (2008). Manual of diagnostic tests and vaccines for terrestrial.

[B14] Piasecki T, Chrząstek K, Wieliczko A (2012). Detection and identification of Chlamydophila psittaci in asymptomatic parrots in Poland. BMC. Vet. Res.

[B15] Pickett MA, Everson JS, Clarke IN (1988). Chlamydia psittaci ewe abortion agent: complete nucleotide sequence of the major outer membrane protein gene. FEMS. Microbiol. Lett.

[B16] Read TD, Joseph SJ, Didelot X, Liang B, Patel L, Dean D (2013). Comparative analysis of Chlamydia psittaci genomes reveals the recent emergence of a pathogenic lineage with a broad host range. MBio.

[B17] Sachse K, Hotzel H, Sachse K, Frey J (2003). Detection and differentiation of Chlamydiae by nested PCR. PCR detection of microbial pathogens.

[B18] Sachse K, Laroucau K, Hotzel H, Schubert E, Ehricht RandSlickers P (2008). Genotyping of Chlamydophila psittaci using a new DNA microarray assay based on sequence analysis of ompA genes. BMC. Microbiol.

[B19] Schachter J, Stephens RS (1999). Infection and disease epidemiology. Chlamydia: intracellular biology, pathogenesis, and immunity.

[B20] West A (2011). A brief review of Chlamydophila psittaci in birds and humans. J. Exot. Pet. Med.

[B21] Zhang YX, Morrison SJ, Caldwell HD, Baehr W (1989). Cloning and sequence analysis of the major outer membrane protein genes of two Chlamydia psittaci strains. Infect. Immun.

